# Minor Surgery

**Published:** 1889-08

**Authors:** D. L. Peeples

**Affiliations:** Navasota, Texas


### MOLUSCUM FIBROSUM.

BY D. L. PEEPLES, M. D., NAVASOTA, TEXAS.

CASE I.—Mr. S. L., of this place, æt 30, occupation, commercially engaged, health good, habits comparatively good, aspect medium size, and becoming to sanatas, [?] but the victim of an innocent fibromatous tumor, situated in the dextral infra-axillary region between the sixth and seventh ribs. Mr. L. consulted me in regard to its removal, and I advised him accordingly.

Clinical history: About two years ago he discovered a kind of pedunculated nucleus, or cytoblast, which from thence gradually enlarged without pain, until just previous to its exsection, when he began to realize some uncomfortable sensibility from the growing tumor.

### OPERATION.

A solution of hydrochlor. cocaine (4 per cent.) was applied over the parts with absorbent cotton (instead of hypodermically), and then a very superficial incision sufficiently long, from behind forward, was made. Solution of cocaine reapplied in same manner, until anesthesia occurred, and the incision then carried down to the tumor; at this time I grasped it with forceps, and dissected the parts away until it was detached from the subcutaneous connective tissues.

Treatment: The cavity was then cleansed thoroughly with a bichloride solution 1 to 1000, the parts brought together and held in position by means of isinglass plaster, then a small piece of absorbent cotton laid over the wound, and a bandage encircling the trunk to hold the cotton in place. The wound healed by first intention, without any plastic extravasation, and no inconvenience to patient, with exception of local soreness, when the muscles were exerted in the axillary region. A little mild chlor. mer., was administered just after the operation, and quinine continued for two or three days. Patient did not take his bed, though the tumor was quite insignificant, as it was only one inch long and three-fourths of an inch in diameter, after having emptied its contents, the serosity.

TREATMENT OF CIONIS. [?]

CASE II. Mr. J. McC., æt 25, of this place, the prey of a continual annoying hacking cough, for months, with paroxysmal attacks of dyspnœa. He complained of sore throat and severe tickling therein. On examination I discovered a marked case of staphylo-diolysis, producing epiglottic titillati, and laryngo irritatio. At times the wound would rest upon the base of the tongue, at others upon the epiglottis, and probably upon the glottis. In this condition I determined on staphylotomy. Every thing being ready, the uvula was permitted to fall between the blades of a Tieman's uvulatome, the spring touched, amputating the lower half of the uvula, and giving almost instant relief to the cough (or tussis et titillatio), though the parts remained irritated and hyperæmic for several days. I would here advise my cotemporaries of the uselessness in hesitancy concerning the amputation of a troublesome or elongated uvula. The operation is simple, and can be performed with scissors and forceps, where you haven't an uvulatome at hand. This little organ or cellular process, is of little, if any importance, "its use is not clear," hence one can do as well without, as with it."

